# Minimally invasive plate osteosynthesis (MIPO) for distal humeral fractures: a cadaveric study and first clinical application

**DOI:** 10.1186/s12891-023-06189-0

**Published:** 2023-03-18

**Authors:** Valeska Hofmann, Julian Diepold, Mohamed Moursy, Marco T. Birke, Christian Deininger, Florian Wichlas

**Affiliations:** 1 Department of Traumatology and Reconstructive Surgery, Eberhard Karls University Tübingen, BG Trauma Center Tübingen, Tübingen, Germany; 2 No Limit Surgery, e.V., Salzburg, Austria; 3 Department of Orthopedics and Traumatology, Paracelsus Medical University Salzburg, Salzburg, Austria; 4 Institute of Anatomy and Cellular Biology, Paracelsus Medical University, Salzburg, Austria; 5 Institute of Tendon and Bone Regeneration, Spinal Cord Injury & Tissue Regeneration Center Salzburg, 5020 Salzburg, Austria

**Keywords:** Distal humeral fractures, Cadaveric study, MIPO technique, Articular fracture

## Abstract

**Background:**

The indication for minimally invasive plate osteosynthesis (MIPO) may include articular fractures depending on the fracture pattern. The goal of this study was to evaluate the feasibility of the MIPO technique for extra- and intra-articular distal humeral fractures.

**Methods:**

The feasibility of the MIPO technique was assessed on 8 cadaveric elbows and 2 clinical cases. The four surgical approaches tested included a 20-mm ulnar incision, a 20-mm dorsoradial incision, and two incisions for olecranon osteotomy (A and B). Surgical incision A was 40 mm on the osteotomy level of the olecranon, and surgical incision B was an extension of the radial incision toward the osteotomy of the olecranon (80 mm).

The four approaches were tested on 4 extra-articular (AO 13 A3) fractures and 4 intra-articular (AO 13 C3) fractures.

**Results:**

Reduction and plate fixation of all distal humeral fractures (8 cadaveric) with and without osteotomy was feasible. However, when using approach B, the soft tissue tension is reduced due to the wider incision. Nevertheless, both approaches A and B showed the same adequate intra-articular fracture control and reduction.

**Conclusion:**

The MIPO technique for reduction and plate fixation in distal humeral fractures is feasible.

**Level of evidence:**

As a feasibility study, this study cannot be clearly classified into a level of evidence. It corresponds most closely to level IV.

## Introduction

Minimally invasive plate osteosynthesis (MIPO) was first described for distal femoral fractures in 1997 [1]. The control of bone alignment was confirmed by fluoroscopy and rotation clinically. Furthermore, MIPO is commonly applied for fracture stabilization in long bones, resulting in good clinical outcomes in femoral and tibial fractures [2, 3]. Indirect reduction and percutaneous fixation of the plate in MIPO protect the blood supply of the bone and the fracture hematoma and therefore seem beneficial for bone healing [4, 5]. In one of the earliest trials on MIPO for intraarticular distal tibial fractures, improved results for plate osteosynthesis were reported with only minimal wound-healing complications compared to the standard open reduction and internal fixation [6]. However, as extensive detachments of soft tissues and fracture exposure are omitted, an exact preoperative imaging technique and understanding of the fracture are crucial for the application of the MIPO technique. The possibilities of modern diagnostics enable precise fracture analysis and preoperative planning for osteosynthesis; thus, the indications for MIPO are becoming increasingly broad [7, 8].

Distal humeral fractures are typically treated using the dorsal approach. Several variations of this surgical approach have been described [9], and all utilize an extensive incision on the dorsal aspect of the elbow. Increased soft tissue dissection and longer duration of surgery are the result. Hence, it is tempting to hypothesize that MIPO in distal humeral fractures is beneficial to avoid these disadvantages of the dorsal approach. At present, there are no studies on the MIPO technique on the intraarticular distal humerus.

In this respect, we tested the feasibility of the MIPO technique for distal humeral fractures. We determined its applicability for extra-articular and intra-articular distal humeral fractures and for the latter using a minimally invasive olecranon osteotomy. We investigated a suitable incision to insert the plates and to align the shaft fracture and the intraarticular fracture anatomically. Furthermore, the plate position was evaluated postoperatively. We used a perpendicular plate position and compared the correct plate position with the AO manual. After reduction and fixation, X-ray control was performed to control the result. In addition, the ulnar and radial nerves and the collateral vessels were visualized by opening the surgical area to assess their integrity.

## Materials and methods

### Cadavers and patients

The feasibility of the MIPO technique was assessed on 10 elbows, including eight elbows from four Thiel-fixed and Thiel-conserved [10] cadavers (two males, two females; average time of death: 76 years) and two clinical cases with an extra-articular fracture (AO 13 A3).

Four extra-articular (AO type 13-A3) and four intra-articular distal humerus fractures (AO type 13-C3) were created in the cadaveric elbows. A short skin incision was made on the flexor side, and the fractures were produced with a chisel under fluoroscopic guidance. The 4 intraarticular (AO 13-C3) distal humeral fractures were reduced using an olecranon osteotomy via two different approaches, A and B, as described below. The 2 clinical cases involves AO 13 A3 fractures from a 57-year-old male (right side) and a 64-year-old female (left side).

Cadavers were placed in the supine position. The arm was bent over the chest to the contralateral shoulder and stabilized with a roll at 90° flexion of the elbow joint. The elbow was completely mobile during the entire procedure. Bony landmarks (olecranon, radial head, medial/lateral epicondyle and cubital tunnel containing the ulnar nerve) were marked with a waterproof pencil. The 2 clinical cases were operated on in the prone position with the elbow bent over a roll at 90°. An inflatable tourniquet was used for surgery, and the anatomic landmarks, the fracture, and incisions were marked with a waterproof pencil.

### Minimally invasive approaches

Four surgical approaches were developed to allow plate positioning and to control fracture reduction: an ulnar approach, a radial approach, and two approaches for olecranon osteotomy (A and B).

**Fig. 1:**
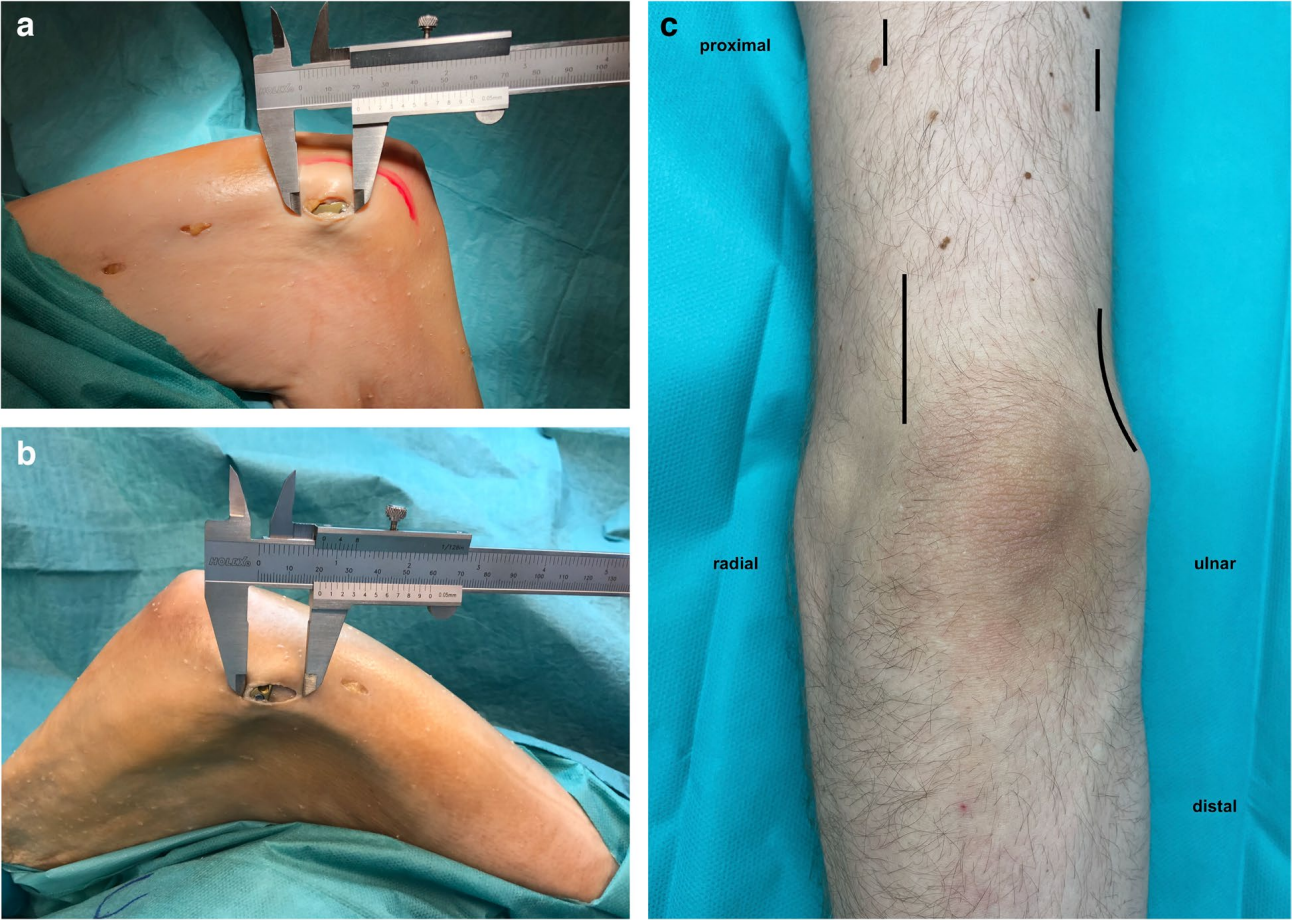
Minimally invasive approach. **a** Ulnar approach (ulnar nerve marked in red). **b** Radial approach via a dorsal incision over the radial column. **c** Schematic drawing of both approaches

The ulnar approach involved an incision performed directly over the fracture extending to the medial epicondyle with a length of approximately 20 mm (Fig. 1a).

The radial approach (Fig. 1b) was dorsally performed over the fracture on the lateral column extending distally with the same length to provide 90-degree plate alignment of the ulnar and radial plate (Fig. 1c). The triceps muscle was mobilized to the medial side.

**Fig. 2:**
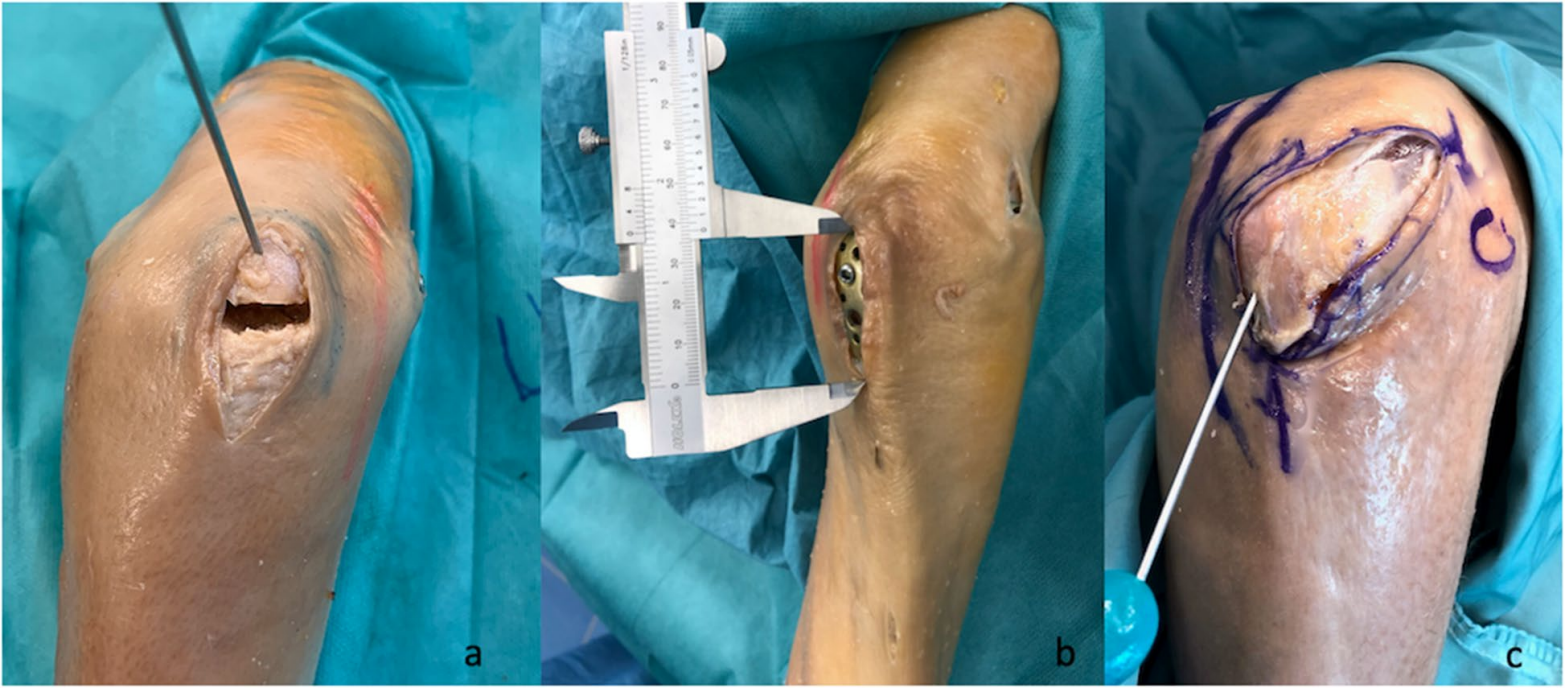
Approaches for olecranon osteotomy. **a** Approach A of the olecranon osteotomy with a chevron osteotomy and a joystick K-wire in the proximal fragment. **b** Length of approach A after osteosynthesis. **c** Skin incision of approach B

To reduce a C fracture correctly under visual control, an olecranon osteotomy is often necessary. For the minimally invasive olecranon osteotomy, we employed two different approaches, A and B. Approach A was located centrally over the olecranon at the level of the osteotomy (length 4 cm). Chevron-type osteotomy was performed, and the proximal fragment was reflected with a joystick K-wire at the tip of the olecranon to open the plane of the osteotomy (Fig. 2a+b).

Approach B was an extension of the radial approach, which was already directed toward the olecranon. Thus, the incision could be easily extended toward the olecranon up to the level of the osteotomy (length 8 cm). The osteotomy was performed as described above (Fig. 2c).

The reduction was controlled digitally and visually through the incisions and through the osteotomy for C3 fractures. For reduction of the extra-articular fractures, we placed two K-wires (2.0 mm) in the distal part of the humerus at the tip of the epicondyles. After fluoroscopic control of the reduction, the K-wires were advanced into the proximal part of the humerus to fix the fracture temporarily. In some cases, a reduction clamp was used over the incisions to facilitate reduction.

After temporary fixation of the fracture, the plates were inserted through the approaches on the bone. We used distal humerus locking compression plates (3.5 mm/2.7 mm LCP; DePuy Synthes, Oberdorf, Switzerland) for 90-degree double-plate osteosynthesis. Here, one plate was placed ulnarly, and one was placed posterolaterally. After temporary fixation of the plates to the bone with K-wires (1.6 mm) through drill sleeves, the position of the plates was confirmed radiologically. Then, the distal screws were inserted over the incision, and the proximal screws were inserted percutaneously through stab incisions into the plate for definitive fixation.

For intra-articular fractures, olecranon osteotomy permitted visual control of the articular surface. Reduction clamps and K-wires were inserted through the existing incisions. The osteotomy was fixed using an olecranon LCP (3.5 mm, DePuy Synthes) that was slid distally under the skin and locked percutaneously distally and through the incision proximally.

**Fig. 3:**
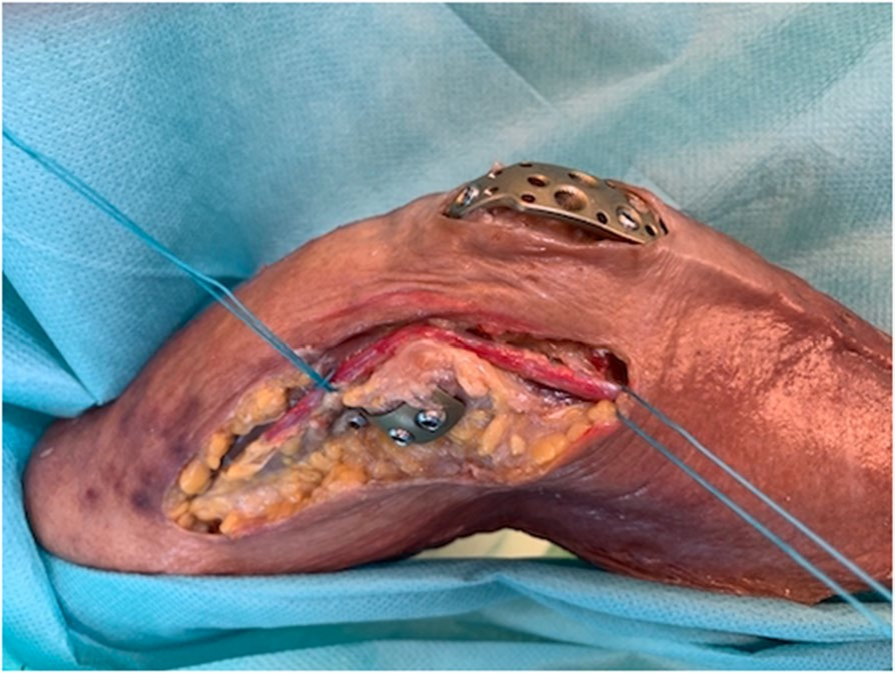
Dissected cadaveric elbow after MIPO of an intraarticular distal humeral fracture using approach A. Ulnar humeral plate, olecranon plate, and the ulnar nerve after dissection of the soft tissues after osteosynthesis (ulnar nerved marked in red)

After radiological assessment of the 8 elbows with the C-arm in 2 planes, the approaches were extended, and the soft tissues were dissected to evaluate the plate positions and to detect possible injuries to the soft tissues, particularly the ulnar nerve (Fig. 3). The AO Manual was used as a reference for the plate position. Attention was given to the bone-plate distance and the correct orthograde alignment of the plate.

We evaluated the overall feasibility of the reduction and fixation of the 8 cadavers and 2 patients using the tested approaches, including the ulnar, radial, and osteotomy A and B approaches. In this respect, we assessed the reduction and plate positioning of all 8 elbows radiologically and after dissection of the 8 cadaveric elbows. Soft tissue damage of the cadaveric elbows was controlled in regard to unforeseen damage and ulnar nerve injury.

The reduction and control of the fractures and the application of percutaneous K-wires and small and large reduction clamps were tested. Osteotomy approaches A and B were compared.

In the two clinical cases with AO 13-A3 fractures, we exclusively used the ulnar and radial incisions. An olecranon osteotomy was not needed, so approaches A and B were not employed.

## Results

The reduction and plate fixation of all distal humeral fractures (4 cadaveric AO 13-A3 fractures, 4 cadaveric AO 13-C3 fractures, 2 AO 13-A3 fractures in patients) with and without osteotomy was feasible.

**Fig. 4:**
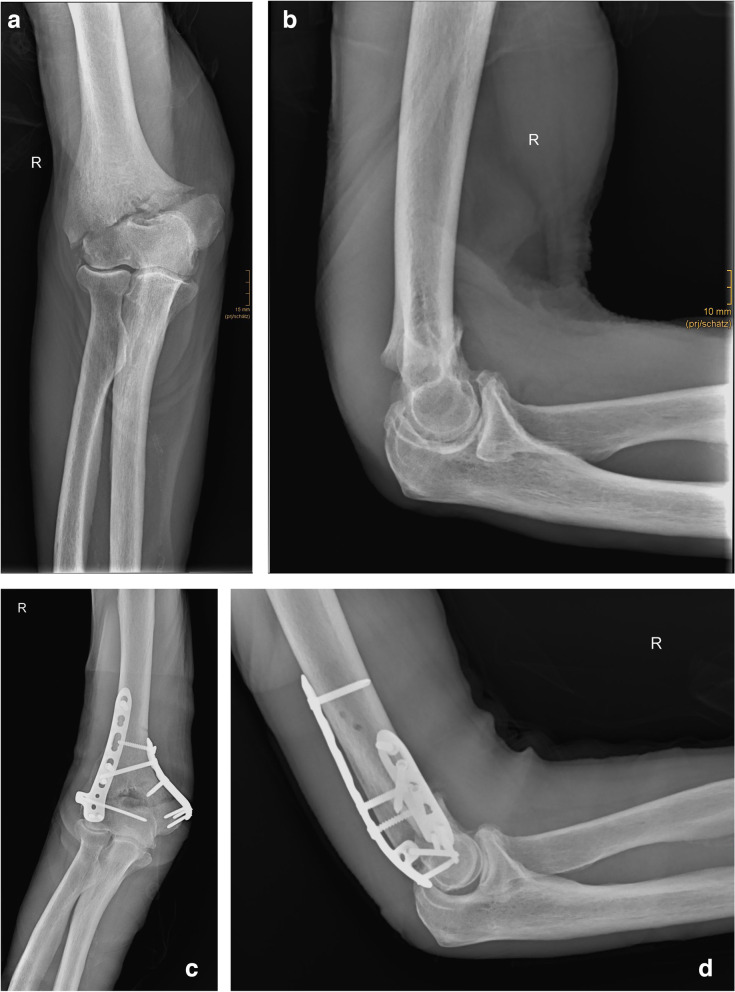
Extraarticular fracture of the distal humerus (AO 13 A3) in a patient. **a,b** Preoperative X-ray shows distal humerus fracture AO 13 A3. **c,d** Postoperative X-ray control

Insertion of the plates on the humerus, temporary K-wire fixation through the ulnar and radial approaches of the plates and temporary fracture fixation percutaneously, and screw placement were possible in all 8 cadaveric distal humeral A3 and C3 fractures as well as the 2 A3 fractures in patients without obvious pitfalls (Fig. 4). The radiological control showed adequate fracture reduction, plate, and screw positioning in both planes for all ten distal humeral fractures.

The dissection of the cadaveric elbows showed adequate fracture reduction and plate positioning and did not reveal undue soft tissue injury. On the one hand, we examined the joint surface clinically. No steps or gaps were noted in any of the specimens. The fragments could be reduced anatomically with good bony contact. We also examined the fracture position with regard to the axial alignment of the humerus. Here, no clinically relevant deviations with regard to rotation or varus and valgus were found. The ulnar nerve was not injured macroscopically in all cadavers. The radial nerve also showed no injury macroscopically at the proximal incision. The 2 patients’ postoperative courses were uneventful. No neurologic deficits, vascular injuries or wound infections were noted. The range of motion was satisfactory in the last clinical controls. Both patients showed free pro- and supination at 6 months, and only one showed limited extension of 10 degrees in the elbow joint with free flexion.

**Fig. 5:**
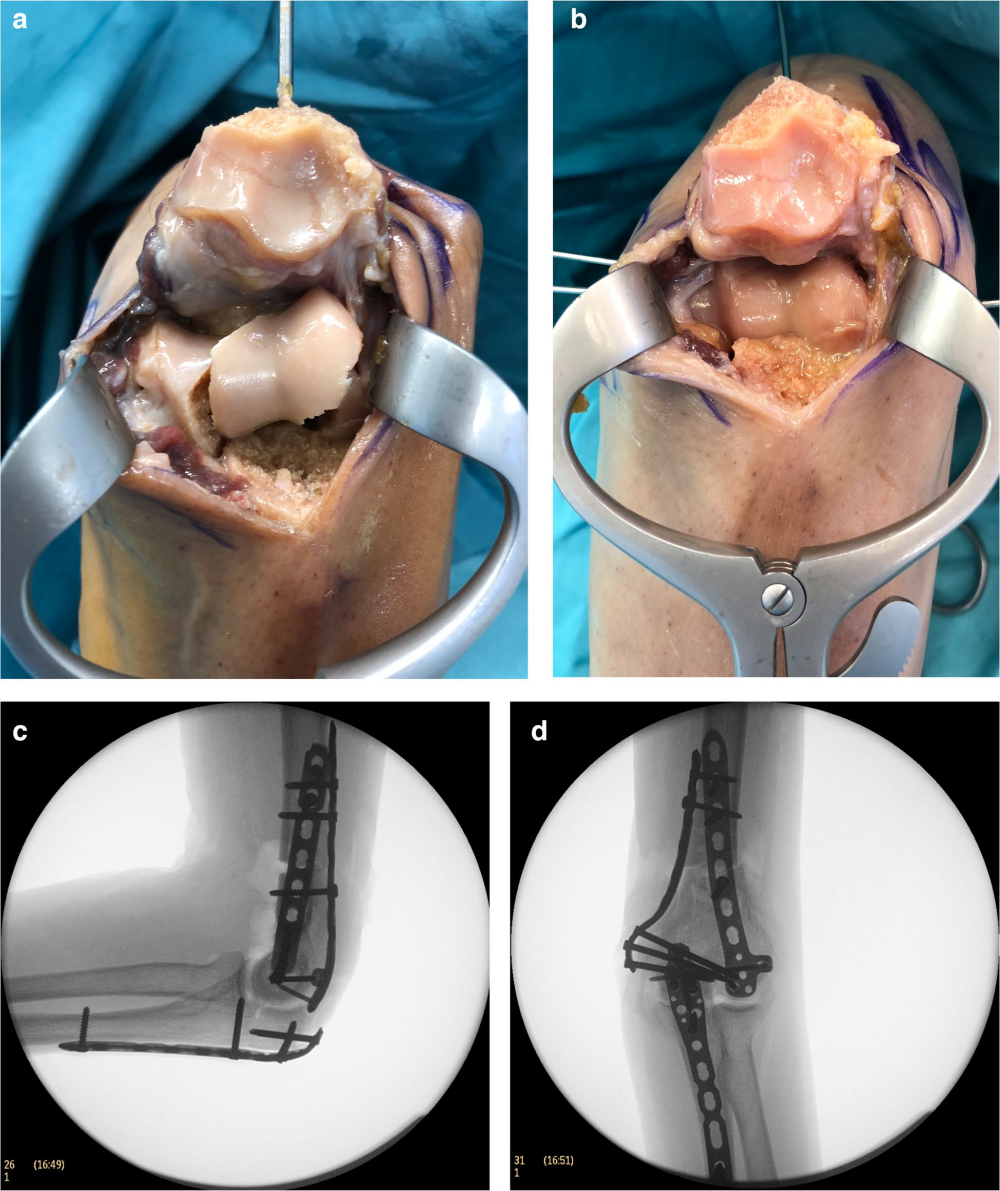
Intraarticular fracture of the distal humerus (AO 13 C3) in a cadaveric specimen. **a** Approach B of the olecranon osteotomy with view on the articular surface of a fractured distal humerus. The approach is too extensive to be called MIPO. **b** Same as **a** after reduction. **c,d** Anteroposterior and lateral intraoperative X-ray after osteosynthesis with double plating of the humerus (3.5 mm ulnar and 3.5/2.7 mm radial LCP) and fixation of the osteotomy with olecranon LCP (3.5 mm LCP)

Reduction and control of all fractures, including intra- and extra-articular fractures, could be performed without notable events using the ulnar and radial approaches as well as both osteotomy approaches A and B. It was possible to see the fracture landmarks sufficiently on the humeral metaphysis using both approaches (ulnar and radial) and those in the articular surface through the osteotomy (Fig. 5), thereby ensuring adequate control on the reduction of the fracture. The application of K-wires and the positioning of reduction clamps was possible using the existing approaches (ulnar, radial, osteotomy A and B).

Both osteotomy approaches allowed safe osteotomy of the olecranon, sufficient manipulation of the fractures, and control of the articular surface of the distal humerus. Although approach A had an incision length of only 40 mm, approach B permitted easier reduction because of the lower tension of the soft tissues. If the overview of two small incisions as described in approach A is not sufficient, this can be improved via an extension to approach B. Nevertheless, the radial approach was already included in approach B. For approach A, the sum of the length of all incisions was 80 mm (20 mm ulnar, 20 mm radial, and 40 mm osteotomy) (Fig. 2b). For approach B, the sum of the lengths was 100 mm (20 mm ulnar and 80 mm radial + osteotomy) (Fig. 2c). The reduction and plate fixation of the osteotomy could be accomplished with both approaches, including screw insertion.

Although approach B provided better view and control of the articular surface, approach A was less extensive. The lengths of the two approaches were measured, revealing a difference in length of 20 mm (100 mm compared to 80 mm) (Fig. 2b/c). Nevertheless, we found no essential differences regarding control and reduction of the articular surface or the osteotomy between approaches A and B.

## Discussion

The MIPO technique has gained increasing popularity for the treatment of humeral fractures [7, 11] and has already been established for use in proximal humeral fractures [12, 13]. An important prerequisite for the application of this technique is the possibility for sufficient fracture control, fracture reduction, and correct plate positioning. The results of this study show that these requirements can be fulfilled to allow the use of the MIPO technique for the treatment of extra- and intra-articular distal humeral fractures.

The standard surgical exposure for distal humeral fractures is the dorsal approach with an extensive incision and a full thickness flap. Different variations of the approach, especially for its dissection through or around the triceps muscle to the bone, exist: the paratricipital approach [14], the modification according to Newcastle [15], the triceps splitting approach and its modification [16]. To preserve muscle integrity, the triceps reflecting approach was established [17] and modified to the “triceps-reflecting-anconeus-pedicle” approach (TRAP) [18]. The most common approach for intraarticular fractures of the distal humerus is the dorsal approach with olecranon osteotomy [19]. Each approach described has its advantages and disadvantages, but there seem to be no differences in the functional outcomes [20, 21]. The many different approaches may indicate that an ideal approach for the treatment of distal humeral fractures has not yet been established. However, an extensive incision is necessary for any dorsal approach described thus far [9]. The duration of surgery and the length of the incision correlate directly with the wound infection rate [22]. In this study, the length of the incision was kept as short as we thought possible. In the two clinical cases, it was possible to minimize soft tissue damage by making smaller skin incisions.

The abovementioned reports describe the general feasibility of the MIPO technique and highlight its advantages and disadvantages. A clear advantage of small approaches used in the MIPO technique is the reduced damage to the blood supply of the bone and to the soft tissues compared to standard surgery. Our dissections of the cadaveric elbows after surgery showed no unexpected damage to the soft tissues or the ulnar nerve. Furthermore, the muscles remained macroscopically unharmed; this finding is in contrast to other studies in other anatomic regions, such as in the proximal humerus [23]. In their study, the authors described a partial lesion of the distal deltoid insertion caused by the insertion of the plate on the humeral shaft in the MIPO technique in a cadaveric study.

A possible disadvantage of the MIPO technique discussed in the literature was the fact that it is not possible to see and control the entire fracture and that its reduction can be cumbersome. Nevertheless, adequate fracture reduction can be achieved by controlling a few but crucial landmarks. For extraarticular fractures, the control of the entire fracture plane is not necessary to reduce the fracture or to place the plates. The strategically placed incisions, ulnarly and radially, right on the fracture site, made it possible to control the fracture sufficiently for reduction on both humeral edges. Furthermore, these incisions permitted the adequate positioning of plates onto the distal humerus to fix the fracture. However, plate positioning in the MIPO technique has been discussed as another disadvantage, as small incisions limit the working space and are expected to be more challenging. In our study, adequate plate positioning was confirmed radiologically in all 10 elbows and by dissection of the surrounding tissues in the 8 cadavers.

In addition to the abovementioned advantages of minimally invasive fracture treatment, disadvantages are also noted. In our subjective opinion, the overview achieved by the shorter incisions is sufficient for adequate reduction and insertion of the plates but limited compared to the open procedure. In the elbow area, a large number of vessels and nerves run within a small region. The location of these structures must be precisely known by the surgeon. In addition, due to the small number of cases and the fact that only one surgeon has performed these operations, our results of clinical cases represent a subjective assessment of the applicability of the MIPO technique. Nevertheless, the results of the experiments on the cadaver show that its implementation is feasible.

Clinical studies on the MIPO technique on the distal third of the shaft of the humerus have been reported in the literature [7, 24]. These results have demonstrated that the MIPO technique is feasible in this region of the humerus and achieves good results. However, studies on intra-articular fractures on the distal humerus have not been reported to date.

The use of the MIPO technique for articular fractures has been described only recently for distal tibial fractures [25, 26]. The approaches developed in these studies permit the sufficient control of fractures in the articular surface and plate insertion through a singular limited incision. Similar to our approaches to the distal humerus, proximal locking of the plates was performed percutaneously. Intraarticular fractures of the distal humerus, especially when comminuted, are treated best by olecranon osteotomy. In our experience, a compression screw outside the plate can also be used with this technique, as the preparation is sufficient. In our cases, reduction and stability could be achieved via k-wires and compression plates, so we did not use an additional screw. We expanded our indication for MIPO to intraarticular fractures using olecranon osteotomy with two different approaches. Although approach B was too extensive to be called minimally invasive in our opinion, approach A showed a surprisingly satisfying view and control of the articular surface of the distal humerus. The overview of the articular surface was similar to that of the conventional dorsal approach. Given the smaller incision and less soft tissue dissection, we prefer approach A for the treatment of intraarticular distal humeral fractures. However, when performing incision A, special attention must be paid to the ulnar nerve during further preparation and insertion of the plate. This nerve lies in the surgical area and represents a vulnerable structure. Both approaches A and B allowed the adequate surgical treatment of these fractures.

Limitations of the study include the fact that it was mainly a cadaver study, and only two patients were enrolled. The transferability of biomechanical studies to the in vitro model is not always accurate. Nevertheless, this study is a proof of concept and demonstrates general feasibility. A subsequent clinical study with a sufficient number of patients is necessary to recommend it for clinical application. We think that the development of further MIPO techniques for articular fractures is beneficial for the patient’s outcome and can generate equal osteosyntheses compared with open techniques.

## Conclusion

In this cadaveric study, we were able to show that it is possible to reduce and fix extra- and intra-articular distal humeral fractures with locking plates using the MIPO technique in cadavers and two clinical cases.

## Data Availability

The data supporting the findings of this study are available upon request from the corresponding author (Valeska Hofmann, M.D., M.Sc.; Email: vhofmann@nolimitsurgery.com).
